# Medical Care for Swimmers

**DOI:** 10.1186/s40798-016-0051-2

**Published:** 2016-07-25

**Authors:** Morteza Khodaee, George T. Edelman, Jack Spittler, Randall Wilber, Brian J. Krabak, Daniel Solomon, Scott Riewald, Alicia Kendig, Laura M. Borgelt, Mark Riederer, Vladimir Puzovic, Scott Rodeo

**Affiliations:** 1 Department of Family Medicine, University of Colorado School of Medicine, Denver, CO USA; 2 Edelman Spine & Orthopaedic Physical Therapy, Dover, DE USA; 3 Department of Physical Therapy, University of Delaware, Newark, DE USA; 4 United State Olympic Committee, Colorado Springs, CO USA; 5 Rehabilitation, Orthopedics and Sports Medicine, University of Washington and Seattle Children’s Sports Medicine, Seattle, WA USA; 6 Marin Orthopedics and Sports Medicine, Novto, CA USA; 7 Winter Sports, United States Olympic Committee, Colorado Springs, CO USA; 8 Department of Clinical Pharmacy and Family Medicine, University of Colorado Skaggs School of Pharmacy and School of Medicine, Aurora, CO USA; 9 Department of Pediatrics and Orthopaedic Surgery, University of Michigan, Ann Arbor, MI USA; 10 Faculty of Medical Sciences, University of Kragujevac, Kragujevac, Serbia; 11 Sports Medicine and Shoulder Service, Orthopaedic Surgery, The Hospital for Special Surgery, Weill Medical College of Cornell University, New York, NY USA; 12 Tissue Engineering, Regeneration, and Repair Program, The Hospital for Special Surgery, Weill Medical College of Cornell University, New York, NY USA

## Abstract

Swimming is one of the most popular sports worldwide. Competitive swimming is one of the most watched sports during the Olympic Games. Swimming has unique medical challenges as a result of a variety of environmental and chemical exposures. Musculoskeletal overuse injuries, overtraining, respiratory problems, and dermatologic conditions are among the most common problems swimmers encounter. Although not unique to swimming, overtraining is a serious condition which can have significant negative impact on swimmers’ health and performance. This review article is an attempt to discuss various issues that a medical team should consider when caring for swimmers.

## Key Points

- Swimming training often consists of frequent weekly practice sessions with long hours.
- Musculoskeletal overuse injuries, particularly shoulder injuries, are common among swimmers.
  Medical team should be aware of risk of overuse injuries, nutrition deficits, and overtraining among swimmers.

## Review

### Introduction

Swimming is one of the most popular sports worldwide and is the second largest sport based on the number of athletes at the Olympic Games [1]. More than 219 million people watched the 2012 Olympic Games with swimming being one of the most watched events [2]. At the Olympics, competitive swimming has pool events ranging in distance from 50 to 1500 m (lasting from under 20 s to over 15 min) and official open-water swimming events that can be as long as 25 km (lasting more than 5 h).

Despite its popularity as a sport, there is a lack of large-scale, methodologically sound, comprehensive epidemiological studies on swimming injuries and illness. Mountjoy et al. have recently published a consensus statement on the definition of injury and illness in Fédération Internationale de Natation (FINA), which is the governing body for aquatic sports [1]. In this paper, we attempt to discuss medical care guideline for swimmers not only at elite level but also in a wide array of different competition and age levels [3].

While the focus of this article relates to swimmers, many principles from the guidelines (e.g., pulmonary issues and dermatoses) should be valuable for other aquatic athletes. The available scientific literature and an experienced group of experts on the topic was gathered to provide practical and sound guidelines.

### Physiology of Swimming

Compared to other types of exercises, swimming is unique as it takes place in the water, which results in different gravitational and resistive forces [4]. Horizontal body position also alters the gravitational effect on circulation. Environmental factors such as water temperature have deep impacts on peripheral vascular physiology which ultimately affects the entire cardiovascular system. Respiratory physiology is also unique as breathing is restricted by stroke [4].

As in all activities, the body utilizes three main energy pathways to produce the ATP that fuels muscle contraction and swimming performance [5, 6]. These systems are the phosphocreatine system, the anaerobic glycolytic system, and the aerobic system; details of each are provided in Table 1.

**Table 1:** Energy systems and their characteristics. Reproduced from Salo et al. [7] with permission

	Characteristics
Anaerobic phosphocreatine	• Fuels intense swimming lasting from 0 to 12 s
	• ATP production is limited by amount of creatine-phosphate present in the muscles
	• Generally associated with high intensity, sprint performance (e.g., 50 m)
Anaerobic glycolysis	• Fuels high intensity swimming lasting up to 2–3 min
	• Generally associated sprint and middle distance swimming events (e.g., 100- and 200-m swims)
	• An end product of anaerobic glycolysis is lactic acid
Aerobic	• Fuels performances lasting longer than 3 min
	• The percentage of energy derived from fats and carbohydrates is dependent on swimming intensity—the higher the intensity, the greater the reliance on glucose
	• Generally associated with endurance activities (e.g., 400 m and longer)

While the energy systems are associated with seemingly specific characteristics, in reality, all three systems work in concert to provide a seamless energy continuum that supports all swimming performances, with different systems contributing energy at different levels depending on the event and/or swimming intensity. Table 2 shows the relative energy contributions of each energy system for a range of maximal-effort swimming events.

**Table 2:** Relative energy system contributions to maximal performance in a range of swimming events. Reproduced from Rodrigues et al. [5] with permission

Distance (m)	Phosphocreatine (%)	Anaerobic glycolytic (%)	Aerobic (%)
50	15–80	2–80	2–26
100	5–28	15–65	5–54
200	2–30	25–65	5–65
400	0–20	10–55	25–83
800	0–5	25–30	65–83
1500	0–10	15–20	78–90

It is important to note that the basic premise of training is to challenge the energy continuum so the body can adapt and enhance the way energy is produced and delivered to the muscles. As such, a majority of the training swimmers’ experience is designed to develop the physiological systems that support performance. Event-specific training should be designed to achieve the following goals [5, 6]:

- *Sprint training* (*50 m*): Enhance the capacity to produce energy through the phosphocreatine system and optimize the enzymatic processes associated with this pathway.
- *Middle distance training* (*100–200 m events*): Enhance the ability to produce energy anaerobically, which involves increasing enzymatic activity associated with anaerobic metabolism and improving the body’s buffering capacity, or tolerance, for lactate.
- *Distance training* (*400 m events and longer*): Enhance lactate removal and improve the myriad cardio-respiratory functions and biochemical processes associated with delivery of oxygen to working muscle (i.e., improve VO2max).

An understanding of physiology is not only important for understanding training and competition but also relates directly to technique and how an athlete prepares for and recovers from training [7]. In a recent study, US-based coaches reported routine use of a variety of physiologic and biomechanic monitoring systems (e.g., heart monitoring, lactate monitor, 2D/3D video-based system monitor) [8]. Proper swimming mechanics will improve metabolic efficiency as propulsive forces increase and drag forces increase. Conversely, fatigue brought on by the depletion of ATP can contribute to technique breakdown and injury. Athletes must engage in a warm-up that will “prime” the cardiovascular system and prepare the body for intense training or competition. Athletes also need to warm down appropriately to remove lactate and facilitate recovery. Paradoxically, the shorter the race (and the greater the reliance on anaerobic pathways) the longer the athlete should warm down. General guidelines are to warm down for approximately 20 min at an intensity that gives a heart rate of about 130 beats/min [7].

### Nutrition

Elite swimmers train on average 2–4 h per day but can often exceed 5–6 h. Due to this often variable, high level of energy expenditure, attention to fueling and dietary intake is crucial to maximize the desired adaption from training and to enhance recovery. A periodized nutrition plan that reflects changes in training volume and intensity will support the energy and nutrient needs of the athlete. Increased attention and education is needed to ensure the swimmer achieves appropriate energy balance to prevent Relative Energy Deficiency in Sport (RED-S) which many swimmers are susceptible to, due to their high training volumes and limited recovery times [9]. Common signs and symptoms of RED-S are frequent in swimmers: increased injury risk, impaired immune system functioning, and decreased training response.

Swimmers training in the water two times per day can easily reach total calorie needs of 4000–5000 kcal/day, despite their highly efficient stroke techniques [10]. Manipulation of dietary carbohydrate intake easily adjusts for the changes in training volume and thus energy demands, throughout the season [11]. For sprint swimmers, carbohydrate needs may only be 5–7 g of carbohydrate per kg of body weight, but a distance swimmer may need as high as 10–12 g of carbohydrate per kg body weight daily due to their heavy training loads [11, 12]. Protein needs for most swimmers are often ~1.4–1.8 g protein per kg body weight, with intakes exceeding this amount taking the place of other macronutrients (mainly carbohydrates and often essential fatty acids when caloric intake is limited). The balance of energy needs must then be met from essential fatty acid intake, preferably from plant- and fish-based sources which contain natural anti-inflammatory properties. Adequate daily consumption of nutrient dense foods (whole grains, fresh produce, and lean protein) should be encouraged for swimmers to resiliently adjust to the stress and demands of heavy training. Common micronutrient deficiencies in swimmers are low vitamin D and iron status, which can limit training quality and decrease immune system functioning [9, 13].

### Supplements

The use of dietary supplements in the swimming world continues to grow in popularity. High training volumes, limitations of digestion time, and time constraints due to travel and fatigue from heavy training create a niche for convenience products often found in the dietary supplement form. Decreased regulation of manufacturing standards and quality assurance in dietary supplements (DSHEA Act, 1994) has increased the risk for anti‐doping rule violations and health concerns as potential side effects when athletes are choosing which products to take [14]. Athletes should consult with qualified health and performance professionals before taking any sports nutrition or supplement to confirm safety and efficacy. Table 3 summarizes the three categories of available dietary supplements used in sport.

**Table 3:** Available dietary supplements in sport. Reproduced from the Australian Institute of Sport Dietary Supplement [15] with permission

Category	Function	Examples
Sport foods	Convenient foods used to deliver necessary macronutrients (e.g., carbohydrates and protein) before, during, and after exercise	Protein shakes, sports drinks, gummy chews, chewable tablets
Medical supplements	Tablets, capsules, or liquids of essential nutrients needed to treat diagnosed conditions or deficiencies affecting health and performance	Iron and vitamin D supplements
Performance supplements	Naturally found dietary ingredients in supplemental form taken to ergogenically aid performance and recovery time	Beet juice, caffeine, beta alanine, creatine, sodium bicarbonate

### Common Medical Issues

#### Musculoskeletal

##### Acute Injuries

Although most musculoskeletal problems in swimming are overuse injuries due to chronic overload, there are several acute types of injury that can occur in swimmers. Most truly acute injuries occur due to contact or direct trauma and are thus uncommon in a non-contact sport such as swimming. However, this can happen in swimming due to striking the hand on the wall at the finish of a sprint. Metacarpal fractures requiring surgery have occurred in elite-level swimmers. Hand and finger contusions and fractures may also occur by striking the hand on the lane line or by striking the hand of another swimmer. Foot injuries can occur from striking the foot on the wall during a flip turn. These mechanisms of injury can also lead to lacerations.

Shoulder pain is common in swimming and is typically a chronic injury due to repetitive overuse. However, acute shoulder subluxation can occur in swimmers with underlying shoulder laxity. For example, the overhead position of the arm during hand entry in backstroke can predispose to shoulder subluxation [16, 17]. Swimmers often have some degree of underlying shoulder laxity, and this can predispose to acute subluxation. Patellar subluxation can occur in individuals with underlying generalized laxity and, although uncommon, can occur during breast stroke swimming [17]. Acute meniscus tears are uncommon in swimming, but symptoms from a degenerative meniscus tear in an older athlete may be exacerbated by breaststroke swimming due to the forceful rotatory and valgus loads on the knee [17].

Acute onset of back pain can occur in swimmers. The repetitive hyperextension that occurs in breaststroke and butterfly may lead to spondylolysis. Although spondylolysis is usually a chronic process, the onset of symptoms can be acute [18]. The repetitive trunk motion from flip turns may also lead to fatigue of the core stabilizing muscles, contributing to back pain [18]. Acute onset of back pain is often simply due to fatigue of the lumbar paraspinal muscles resulting from overload during swimming or dryland training. Other less common causes of acute low back pain include disc herniation and facet joint injury.

It should also be recognized that there may be an acute exacerbation of an underlying chronic condition. For example, a swimmer with shoulder tendinopathy (“swimmers shoulder”) may have an acute exacerbation of shoulder pain due to a change in training volume or frequency [16, 17, 19]. A survey of injuries in a NCAA Division I collegiate swimming program reported that freshman had the highest rate of injury, supporting the relationship between a change in training and injury [20]. A careful history is critical to determine if an apparently acute presentation of pain or injury is actually an exacerbation of a pre-existing problem. Acute exacerbation of a pre-existing, chronic injury is rather common.

Swimmers often do weight lifting and other “dryland training” as part of their training program. Acute musculoskeletal injury can occur during these training activities [19]. Acute muscle strain injury can occur with forceful weightlifting or other forms of resistance exercise. Notably, a report of injuries occurring over five seasons in a NCAA Division I collegiate swimming program found that 38 % of all injuries occurred during dryland training [20]. Attention to technique and careful performance of dryland exercises is critical to prevent injury from these exercises. Relative rest of the irritated region and a focus on core strength and rehabilitation of supporting musculature is the key to treatment.

##### Overuse Injuries

Overuse injury risk in swimmers has been reported as high as 4.0 injuries per 1000 athletic exposure (AE) [16, 19–22]. In a study of swimmers at the University of Iowa from 2002 to 2007, the shoulder and upper arm were the most commonly injured areas followed by the back and neck [20]. In the NCAA Injury Surveillance Program study for a 4-year span, the rate of overuse injuries in men was 0.66 per 1000 AE and in women was 1.04 per 1000 AE [22]. In a study of the 2009 FINA world championships, 171 injuries were reported (incidence of 66.0 per 1000 registered athletes) [23]. The most affected body part was the shoulder and the most common cause of injury was overuse [23].

###### Shoulder

Shoulder pain is the most common musculoskeletal complaint in swimmers [20]. The vast majority of shoulder issues in swimmers pertain to overuse rather than from acute injuries (Fig. 1) [23, 24]. Differential diagnosis of swimmer’s shoulder pain is extensive [17]. An appropriate work-up including a detailed history, and thorough physical examination of the neck, shoulder, and upper back is critical to developing and narrowing this differential. The medical practitioner must also be aware of physiologic stressors and biomechanics during the swim stroke similar to how we have come to understand throwing mechanics in baseball. Poor biomechanics, especially with fatigue of scapular stabilizing and rotator cuff muscles, leads to dynamic instability and impingement [19]. A multitude of issues contribute to the “swimmer’s shoulder”: a hypermobile glenohumeral joint combined with technique flaws or fatigue can lead to impingement of the rotator cuff [17]. Hypermobility can be multifactorial: the swimmer may have a predisposition with shoulder laxity or may have a traumatic event. Microtears or stretching of the glenohumeral ligaments exacerbate static instability. Once these athletes fatigue, their rotator cuff cannot alone stabilize the humerus and the increased translation, especially upward or superior-posterior leads to overloading the rotator cuff tendon. Abduction and rotation with humeral head translation can result in contact of the posterior supraspinatus or anterior infraspinatus against the posterior glenoid rim and labrum—a condition also described as “internal impingement.” Fortunately, most of these cases can be treated with rehabilitation and a proper strengthening routine as well as improvement in potential stroke flaws [27]. A group of strength training exercises for rehabilitation of the rotator cuff, scapular stabilizers, and abdominal and low back core muscles is helpful as either preventative or rehabilitative exercises for the shoulder (Fig. 2) [19].

**Fig. 1:**
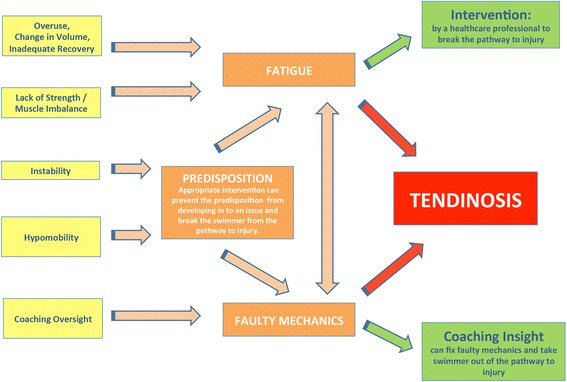
Pathway to injury: competitive swimmer. Reproduced from Edelman et al. [25, 26] with permission

**Fig. 2:**
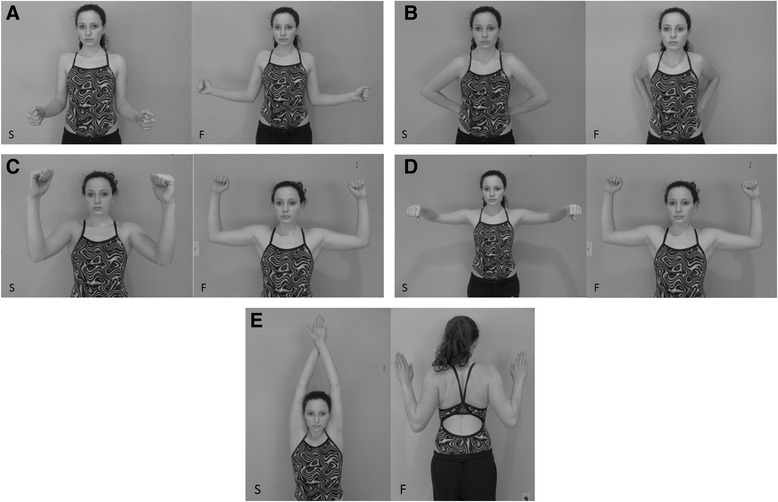
On-deck active warm-up (*S* start, *F* finish). Complete 2 sets of 15 for each activity. Active Warm-Up: **a** Place your arms by your side, bend your elbows to 90° to assume the start position. Externally rotate your arms to the end range at a comfortable pace and then return to the starting position. As you externally rotate, pinch your shoulder blades together. Do not force the end range. Complete 2 sets of 15 repetitions. Active Warm-Up: **b** Place the back of your hands on your back at the belt line and bring your elbows forward to assume the start position. Squeeze your elbows and shoulder blades together and then return to the start position. Complete 2 sets of 15 repetitions. Active Warm-up **c** Forward elevate your arms to 90° and then bend your elbows to 90° to assume the starting position. Horizontally abduct your arms to a “goal post” position, squeezing your shoulder blades together at the same time. Then, return to the starting position. Complete 2 sets of 15 repetitions. Active Warm-up: **d** Abduct your arms to 90° and bend your elbows to 90° to assume the starting position. Then, externally rotate your shoulders to achieve the “goal post” position. Return to the starting position. Complete 2 sets of 15 repetitions. Active Warm-up: **e** Assume a tight streamline for the start position. Drop your elbows into your “back pockets,” while squeezing your shoulder blades together and keeping your hands up. Return to the streamline position and complete 2 sets of 15 repetitions

###### Hip and Groin

Breaststroke, far more commonly than other competitive swimming strokes, can lead to a variety of hip and groin strains and injuries. Adductor strain, iliopsoas strains, and sports hernias pose difficult diagnostic and treatment challenges. Symptoms can be quite similar among these entities. Given the repetitive nature of swimming, these can also be notoriously problematic over an extended time [28]. Stretching and supportive core strengthening will aid in recovery. A narrower breaststroke kick will decrease strain on the hip adductor muscles [29]. This can be facilitated using a number of technique drills in the pool. Prophylactic stretching and strengthening is always better than treating an issue.

###### Knee

Up to 75 % of breaststrokers report medial knee pain [30]. Medial knee pain can occur from strain on the medial collateral ligament, irritation of a medial plica, or, in unusual cases, medial meniscus tears. Patellar instability is another less common differential diagnosis to consider. Examination of the knee should be focused on evaluation of knee stability. Knee injuries from cross-training such as weight-lifting, plyometrics, or running can be exacerbated by swimming. Improving hip flexibility and avoidance of an overly wide breaststroke kick will help decrease strain on these areas of the knee.

###### Lumbar Spine

Dolphin kicking plays an important role in modern swimming technique. With an increased emphasis on this facet of swimming comes risk of low back injury, as hyperextension with dolphin kicking can lead to pars interarticularis pathologies (e.g., spondylolysis). The L5-S1 disc is another common area of irritation with overuse. Similar to other areas of overuse in swimmers, dryland training, especially weightlifting, can exacerbate or cause back injuries [31, 32]. Also, similar to other areas of overuse, a prevention program with emphasis on core abdominal and low back strengthening can help prevent chronic low back complaints.

##### Shoulder Rehabilitation

Changes in training practices and improved research allow healthcare providers to manage shoulder pain in competitive swimmers better than ever before. In the past, the healthcare providers generally attributed shoulder pain to excessive training or too many repetitive overhead strokes leading to laxity and resultant impingement of the rotator cuff [33–37]. Recently, a new treatment paradigm reveals no single cause for shoulder pain in swimmers but rather a cluster of contributing factors that can match the clinical presentation to the treatment [27]. Edelman et al. offers a subclassification system for shoulder pain identifying a cluster of etiological factors: (1) predisposition, (2) fatigue, (3) injury-related (faulty) mechanics, and (4) tendinopathy [27]. This model can guide the selection of homogenous subgroups for more effective treatment strategies as noted in Fig. 1. Identifying individual causes in the setting of this paradigm should allow targeted and more effective treatment.

###### Shoulder Stretching and Dynamic Warm-Up

The shoulder is the most frequently stretched joint among swimmers. To date, swimmers have adopted stretches that target the static stabilizers of the GHJ. In the past, static stretches and pre-race rituals (i.e., ballistic windmill motion) were utilized in hopes of preventing injury [38]. Recent research on swimmer’s flexibility suggests there is no indication that extraordinary shoulder joint motion or flexibility is necessary to achieve a fast, efficient stroke [39, 40]. In addition, there is no evidence that static stretching immediately before activity will reduce muscle injury rates [41, 42]. Many swimmers are generally flexible and possess loose connective tissue (general joint laxity) [43]. Because of their inherent laxity, swimmers should emphasize preserving the overall stability of the shoulder and less time on general static stretches [26]. Instead, a dynamic warm-up (Fig. 2) has been found to produce short-term and long-term performance enhancements in power, agility, strength, muscle endurance, and anaerobic capacity [26, 38, 44, 45]. A dynamic warm-up tends to include some form of dynamic stretching, agility, and plyometric activities and specific motor pattern movements [46]. Figure 2 demonstrates dynamic warm-up which is specifically designed for the swimming population. Swimmers should employ a steady pace with each activity and avoid ballistic type motions.

###### Focused Strengthening Exercises

Dryland training has been an important part of strengthening programs among swimmers at different age and skill levels. The main focus of these training programs seems to be the spine and core strengthening [47]. Strength deficits can play an important role in fatigue development. Madsen et al. found that the majority of swimmers demonstrated signs of scapular dyskinesis in the course of a 100-min swimming session [48]. Through EMG analysis of the painful shoulder, Scovazzo et al. discovered muscle activity of the serratus anterior is significantly depressed through the important pull-through phase in swimmers with shoulder pain [49]. Batahla revealed in the course of a swim season the internal rotators of competitive swimmers become proportionally stronger when compared to their antagonists, increasing muscle imbalance, and the risk of an injury process [50, 51]. Fatigue may lead to a short-term decrease in acromiohumeral distance [52]. As a result, a dryland program focusing on the shoulder external rotators and the scapular stabilizers would be of benefit and should be recommended as routine prophylactic measures [53]. Strengthening the internal rotators should be de-emphasized.

##### Return to Swimming after Injury

If the treatment program results in removing the swimmer from the water, then a careful reintegration back to training is essential [54, 55]. During the rehabilitation process, when the swimmer is able to reach above shoulder height without pain and demonstrate resisted motions from 0° to 90° without pain, then the swimmer is encouraged to return to the pool and swim 1000–2000 m slowly and comfortably while avoiding antagonizing strokes and sprint sets. When the swimmer is without pain during resisted motions in all planes, and during most activities of daily living and swimming 2000 m, then add 500 m every three workouts. During this step, it is indicated to avoid double workouts and sprint sets. When swimming 4000–5000 m without pain, then integrate all four competitive strokes and add short sprint sets.

#### Pulmonary

To ensure the health of swimmers, chlorine-based disinfectants are used to effectively reduce the risk of bacterial and viral infection in swimming pool facilities. However, when chlorine reacts with organic compounds in the pool water (e.g., sweat, urine, soap residues, cosmetics, suntan oil, dirt, and other solid waste material), several volatile chemical compounds are produced in the form of trihalomethanes, chloramines, and haloacetic acids [56]. In turn, exposure to these chemical compounds can occur in swimmers via three direct avenues: (a) ingestion of pool water, (b) inhalation of airborne chlorine and chlorine analogs in the air space located immediately above the pool surface, and (c) direct skin contact with the pool water [57]. Exposure to trihalomethanes, chloramines, and haloacetic acids can have a negative impact on general health and athletic performance. Specifically, there is strong evidence suggesting that acute and/or chronic exposure to these chlorine-based chemical compounds can manifest as clinical symptoms of upper respiratory dysfunction (chronic rhinitis, sneezing, irritated nasal sinuses, runny nose, nasal obstruction, and sinusitis) [58, 59] and lower respiratory dysfunction (breathing difficulty, wheezing, cough, chest tightness, and abnormal spirometry) [58, 60, 61], as well as eye irritation and headache [62]. The negative effects of exposure to chlorine-based chemical compounds on pulmonary function appear to be due to pathophysiological mechanisms related to lung epithelial tissue perturbation [63–65] and airway remodeling [66, 67]. Thus, swimmers face a tough balancing act between the positive effects of a contaminant-free pool and the negative aspects of over-exposure to chlorine and chlorine analogs. Table 4 summarizes the recommendations for potential strategies to control the negative effects of swimming pool chlorine on general health and performance.

**Table 4:** Recommendations to athletes, coaches, and medical staff as potential strategies for controlling the negative effects of swimming pool chlorine on general health and performance

Schedule training sessions in outdoor pools as much as possible where natural ventilation helps to reduce surface chlorine levels. If forced to use an indoor pool, avoid older pools that may have low ceilings and poor ventilation systems. In addition, deck-level industrial fans can be effective in enhancing the clearance of surface chlorine in older, poorly ventilated pools.	
Undergo evaluation of pulmonary function by a certified medical professional for the purpose of determining asthmatic response and/or susceptibility to airway hyper-reactivity in a chlorinated environment [57, 67].	
Consider prophylactic use of antioxidant supplementation (beta-carotene, vitamin C, vitamin E) to reduce the negative effects of chlorine-induced oxidative stress on pulmonary function [69].	
Train in a pool where the professional maintenance staff is conscientious and precise in their regulation of optimal chlorine levels, as well as water temperature and ventilation of surface chlorine.	
Train in a pool where the professional staff ensures that proper swimming pool behavior, etiquette, and equipment are in place to help reduce the level of chlorine exposure. Posted requirements should be in place regarding pre-entry showers, bathroom breaks, suntan lotion, swim caps, goggles, etc.	
Encourage swimming pool maintenance staff to stay current on the latest information regarding the efficacy of potential substitute disinfectant methods such as electro-physical systems (electrolytic copper and electrolytic silver), ozone disinfection, and ultraviolet disinfection [56].	

One study reported that about 20 % of elite swimmers (FINA world championships and Olympic Games) had asthma/airway hyperresponsiveness [68]. In this study, swimmers in comparison to other aquatic athletes had higher prevalence of asthma. This seems to be more related to the endurance nature of swimming and not necessarily due to chlorines [68].

Exercise-induced bronchospasm (EIB) is more common among swimmers in comparison to general population [68, 69]. This may be partially due to more participation as a result of a belief that breathing the warm and humidified air benefits swimmers with asthma and EIB [17]. However, exposure to aerosol chemicals may trigger these conditions [17]. Management of swimmers with asthma and EIB is similar to other athletes [17, 70]. Finally, along with EIB, assessment of exercise-induced laryngeal obstruction (EILO) should also be included in the medical workup of swimmers who present with chronic pulmonary issues [71].

#### Ear, Nose, and Throat

Allergic and non-allergic rhinosinusitis is common among swimmers [72]. Due to easy spread and possible other factors (e.g., suppressed immune system), viral upper respiratory infection (URI) is more common among swimmers than other athletes [17, 70]. Swimmers with symptoms and signs of more serious infections (e.g., pneumonia, fevers, and hypoxia) should not be allowed to practice.

Prolonged exposure to water and frequent attempt to clean the ear canal predispose swimmers to develop acute otitis externa (swimmer’s ear). Gram-negative bacteria, most commonly pseudomonas, are the major pathogens. Treatment with otic anesthetic/anti-inflammatory/antibiotic/acidifier agents is usually adequate [73]. In rare cases with systemic signs (e.g., fever), oral antibiotics may be necessary [73]. Other less common ear problems in swimmers include otomycosis, exostoses (“surfer’s ear”), traumatic tympanic membrane perforation, otitis media, and barotraumas of the inner ear.

#### Dermatologic

Swimmers are susceptible to different dermatologic conditions given their substantial amount of time spent in the water and in moist environments [74]. Certain dermatoses are infection or organism-related. Swimming pool granulomas (atypical Mycobacterium infection) may occur overlying bony prominences and are often found in the upper extremities, especially the fingers. Nodules or plaques arise about 6 weeks after exposure [75]. The affected area should soak in warm, clean water for 5–10 min, three to four times per day. Antibiotics (clarithromycin, minocycline) may also be used [76]. “Hot tub” (*pseudomonas aeruginosa*) folliculitis usually appears within 8–48 h of exposure to contaminated water. Pale green fluorescence under Wood’s lamp can aid in diagnosis [77]. Most eruptions self-resolve within 2 weeks, but acetic acid 5 % compresses may provide symptomatic relief [78]. Deep bacterial folliculitis (“bikini bottom”) may arise in swimmers who wear wet, tight-fitting swimsuits for long periods of time. Maceration of the skin allows for infection with *Streptococcus* or *Staphylococcus aureus*. This can be usually treated with a course of cephalexin or appropriate MRSA coverage, if suspected [79]. Swimmers are also particularly vulnerable to other common skin infections such as molluscum contagiosum (“water warts”), tinea pedis (“athlete’s foot”), and plantar warts [74].

Other swimming-related dermatoses may be caused by irritants or allergies. Goggles are often an offending agent, caused by reaction to the foam, rubber, or plastic lining [80]. A short course of medium-potency topical steroid or an oral steroid burst for swimmers with severe symptoms may be used [74]. Chlorinated or brominated pool water may cause an allergic or irritant contact dermatitis. This can be treated by attempting to avoid the chemical irritant or by typical contact dermatitis care (i.e., moisturizers, topical corticosteroids).

Frictional dermatitis may occur from rubbing the chin on the shoulder when turning the head to breathe or with recurrent contact on rough pool surfaces with hands/feet (“pool palms”) [81, 82]. Lesions generally self-resolve, but petroleum jelly may help alleviate symptoms. Finally, it is important to note that swimmer’s may be at an increased risk for skin cancers, such as melanoma. This is due to sun exposure with the effects possibly potentiated by chlorine [83]. Therefore, it is important to counsel outdoor swimmers on adequate use of water-resistant or water-proof sunscreen.

#### Ophthalmologic

The most common ophthalmologic issue encountered by swimmers is eye irritation and redness related to chemicals contained in swimming pools. This is more commonly experienced in pools containing chlorine or bromine compared to those using ozone, UV, and salt electrolysis treatments [84]. Wearing swim goggles significantly decreases the risk of developing eye irritation [85]. In addition to peri-orbital skin irritation, some studies have shown a small but significant transient increase in intraocular pressure (IOP) in individuals wearing certain types of swimming goggles. Goggles with smaller surface areas lead to larger IOP measurements [86]. Despite some speculation that repetitive goggle use over time could consequently lead to development of glaucoma, one recent study challenges this notion [87]. Another ophthalmologic issue in swimmers is ocular infection. Swimmers wearing soft contact lenses are at risk of developing pseudomonas infection and acanthamoeba keratitis [88, 89]. Acanthamoeba infection is rare but serious. Symptoms may include eye pain/redness, blurred vision, light sensitivity, foreign body sensation, and excessive tearing. Suspected patients should be referred to an ophthalmologist immediately for evaluation, as early detection is essential [88]. Treatment usually consists of topical agents; however, sometimes oral itraconazole therapy and/or surgery may be required in advanced cases [88]. Prevention consists of contact lens removal during swimming or thorough disinfection of lenses following swimming.

#### Neurologic

The main concerning neurologic condition for swimmers is epilepsy. Having a seizure in the water could result in serious immersion-related injury and ultimately drowning. Therefore, it is widely accepted that swimmers with epilepsy should always be supervised while swimming [90]. This is especially important in those athletes with poorly controlled seizure disorders. If an athlete does have a seizure in the water, it is important to extricate them to land as quickly as possible to minimize immersion injury and practice standard seizure care such as body injury prevention and airway management. Even after apparent full recovery, near drowning victims are at risk of major delayed complications, such as secondary drowning [91]. Therefore, all athletes who experience a seizure in the water should be evaluated and monitored at a medical facility.

Often swimmers (more common among synchronized swimmers) will train by holding their breath for extended periods of time in effort to increase respiratory capacity. This may be dangerous as it can result in loss of consciousness and potentially anoxic brain injury [92]. Swimmers should be counseled that these “blackouts” can be dangerous and possibly result in permanent damage.

#### Concussion

Though uncommon, the recognition and appropriate treatment of the concussed athlete is essential in preventing long-term sequelae. The incidence of concussions vary for high school (men = 0.1 per 10,000 AE/women 0.2 per 10,000 AE) [93], collegiate (men = 0.6 per 10,000 AE/women 0.1 per 10,000 AE) [22, 94], and international levels (0.04 per 10,000 athletes). Concussion may occur from a variety of reasons (e.g., when a backstroke swimmer strikes the wall, runs into another swimmer while entering in the water or collision with the ground) [93]. Symptoms are similar to other sports (i.e., headache, nausea, vomiting, and dizziness), and any athlete suspected of a concussion should be removed from practice/competition [95]. Successful management of the concussed swimmer requires coordination of services by a sports medicine provider and team experienced in concussion care. Athletes should initiate a return to school program when appropriate and be symptom free prior to the initiation of a return to swimming/dryland training [95]. All athletes should follow a gradual return to play program that incorporates progression of swimming activity while monitoring symptoms [95]. Any athlete experiencing prolonged symptoms, specific deficits, or multiple concussions may require further cognitive testing prior to returning to swimming.

#### Endocrine and Metabolic

##### Menstrual Cycle and Contraception

The prevalence of menstrual disorders in sports like swimming ranges from 16 to 82 % [96–98]. Several etiologic factors may contribute to menstrual disorders including abnormal levels of hormones, luteinizing hormone (LH) pulsatility, inadequate body fat stores, low-energy availability (EA), and exercise stress [9]. Function hypothalamic amenorrhea occurs through reductions in EA which may disrupt LH pulsatility by impacting the hypothalamic hormone gonadotropin-releasing hormone output and alter the menstrual cycle. During extensive training, rapid or significant fat mass reduction may adversely affect menstrual function. Low EA alters levels of metabolic hormones and substrates including insulin, cortisol, growth hormone, insulin-like growth factor-I (IGF-I), 3,3,5-triiodothyronine, grehlin, leptin, peptide tyrosine–tyrosine, glucose, fatty acids, and ketones.

In the past studies, female competitive swimmers not only appeared vulnerable to delayed puberty and menstrual irregularities but the associated hormonal profile was also very different from the hypothalamic amenorrhea described in dancers and runners. Specifically, dehydroepiandrostenedione sulfate and androstenedione, but not testosterone, were higher than average in swimmers compared with controls [96]. Mild hyperandrogenism, rather than hypoestrogenism, was a suggested mechanism for reproductive dysfunction in swimmers. Furthermore, the prevalence of amenorrhea in runners is 24–26 % compared with swimmers at 12 % [99]. This may be related to the emphasis of leanness in running compared with swimming.

Sports performance during various phases of the menstrual cycle has been researched for many years. Evidence-based conclusions have been extremely difficult to reach due to methodological issues (e.g., inappropriate verification of menstrual cycle phase, timing of hormone samples), inter- and intraindividual variability in hormone concentrations, pulsatile secretion of hormones, and small numbers of subjects. Research has not consistently demonstrated significant differences in aerobic capacity, anaerobic capacity, aerobic endurance, or muscle strength in any specific menstrual cycle phase [100, 101]. However, very active or highly competitive swimmers are at risk of developing the RED-S (formerly known as female athlete triad) which is a syndrome resulting from relative energy deficiency that affects many aspects of physiological function including metabolic rate, menstrual function, bone health, immunity, protein synthesis, cardiovascular, and psychological health [9]. In athletes with amenorrhea for over 6 months, bone mineral density (BMD) should be measured with dual X-ray absorptiometry (DXA). Weight gain, with adequate protein and carbohydrate intake, has been the strongest predictor for return of normal menstrual function in college athletes [102].

The use of contraceptives may be advantageous for swimmers who are negatively affected by their menstrual cycle, as they can provide a stable and controllable hormonal environment for training and competition. Furthermore, swimmers may desire minimal or no bleeding/spotting which can be offered with several different forms of contraception. Examples of long-acting reversible contraceptives with high (>99 %) efficacy include intrauterine devices (IUDs) and the implant [103]. Progestin-only IUDs offer the advantage of minimal to no menstrual bleeding. Injectable depot medroxyprogesterone may cause amenorrhea and adversely affect BMD if used long term (>2 years) [104]. Continuous or extended use of combined (containing estrogen and progestin) hormonal contraceptives can be used for the pill, patch, or ring to avoid menstrual bleeding. Instructions for this include continuous use of a monophasic (same amount of estrogen and progestin) contraceptive by using only active pill, vaginal ring, or patch and discarding inactive pills or skipping the ring-free or patch-free week. Some combined hormonal contraceptive pills are packaged to allow for a menstrual cycle every 3 months with 84 active pills and 7 inactive pills. Although an oral contraceptive may be a less desirable form of contraception due to lower efficacy (91 %) and masking low EA and menstrual dysfunction, the use of oral contraceptives has shown no effects on swimming or endurance performance. Therefore, combined contraceptives should be recommended only when there is a medical indication or other more effective contraceptives (e.g., IUD, implant) are contraindicated, not tolerated, or unacceptable to the swimmer.

##### Diabetes Mellitus

Participating in regular physical activity among patients with type 1 and type 2 diabetes mellitus improves blood glucose control, lipid profile, blood pressure, and fitness [105, 106]. Swimmers with diabetes should be able to compete in endurance events granted that they have no complications of their diabetes and are in good glycemic control [105, 107, 108]. When planning medical coverage, it is crucial to be aware of swimmers with diabetes in advance. Medical teams should be prepared to manage unusual but serious circumstances such as hypoglycemia [107, 108]. Close glycemic and dietary monitoring before and during long practice sessions and races is the key to avoid complications [109]. As insulin is on the prohibited WADA list, a therapeutic use exemption is required.

#### Cold- and Heat-Related Illness

Cold- and heat-related illness is greatly impacted by a variety of factors that can affect the swimming athlete. Both occur when there is a thermoregulatory mismatch between heat production from exercise and heat loss. Body heat loss in the swimmer primarily occurs through convection from the interface of the skin and water that is proportional to speed (heat transfer coefficient) and the temperature difference between the skin and water [110]. Other factors impacting heat loss include the design and thermal properties of garments, the environment (air temperature, humidity), athlete illness, and medications [111, 112].

Pool and open-water venues offer unique environmental challenges for the swimmer. Pool venue environments are often well controlled when in a closed setting but can be impacted by weather (temperature, wind, and humidity) when in an open setting. Open-water swimming venues have greater environmental variability (air and water temperature, humidity, and sun exposure) that can fluctuate during the course of a race leading to hypothermia or hyperthermia. FINA guidelines suggest open-water swimmers should only compete when the water temperature is above 16 °C or below 31 °C to minimize the risk of severe hypothermia or hyperthermia, respectively [110]. Additionally, the impact of cold water immersion and voluntary apnea could result in parasympathetic and sympathetic activity resulting in cardiac arrhythmias, pulmonary edema, and potential death.

The evaluation, treatment, and prevention of environmental-related illnesses require a coordinated effort between the athlete, sports medicine providers, and venue managers. Symptoms will vary for hyperthermia (fatigue, fever, chills, elevated temperature, thirst, confusion, and tachycardia) and hypothermia (chills, fatigue, confusion, and bradycardia). All athletes should undergo removal of wet or damp clothing and have a rectal temperature measurement to obtain an accurate core temperature [111]. Athletes with a temperature >40 °C should undergo cold water immersion as soon as possible to normalize core temperature. Athletes with hypothermia should be warmed with heat blankets/air, as appropriate. Any athletes with cardiovascular compromise will require transportation to a local hospital. Preventive strategies include acclimatization, familiarity with the race venue, adequate warm-up time, and proper hydration [111].

### Overtraining

Overtraining (OT) is a condition experienced by many swimmers, especially those competing at the Olympic level [113]. OT is characterized by several physiological, biological, and psychological symptoms with the most obvious ones being (a) a consistent decrement in performance, (b) an inability to train and recover effectively, and (c) a loss of determination and mental focus [114]. OT can be more accurately portrayed as the end-point on a continuum [114], as shown in Fig. 3. Moving from left to right, the first three phases on the OT continuum are reflective of “positive” training and good performance, analogous to green on a traffic light (“proceed immediately”). Next on the OT continuum, separated by a thin line is a phase that typically includes both “positive” and “negative” training/performance, due primarily to a requisite increase in training load for the purpose of advancing the athlete’s fitness level and competitive performance. This block is analogous to yellow/amber on a traffic light (“proceed with caution”). Finally, on the far right of the OT continuum is OT, which is characterized by “negative” training/performance, analogous to red on a traffic light (“stop”). There are several models that have been proposed to explain the underlying biological, physiological, and/or psychological mechanisms of OT. Among the more credible is the “elevated pro-inflammatory cytokine” hypothesis [115–117]. This model proposes that excessive musculoskeletal stress (two or three training sessions per day for several weeks) in combination with insufficient recovery (sleep, nutrition, psychological down time) leads to chronic musculoskeletal inflammation and the release of the following pro-inflammatory cytokines: interleukin-6 (IL-6), interleukin-1-beta (IL-1β), and tumor necrosis factor-alpha (TNF-α). These pro-inflammatory cytokines act on the central nervous system (CNS), hypothalamic-pituitary-adrenal (HPA) axis, and hypothalamic-pituitary-gonadal (HPG) axis, which manifests in several of the clinical symptoms seen in overtrained athletes (e.g., sleep disturbances, elevated stress hormones, perturbed reproductive function) [115–117]. In terms of the prevention of OT, we offer a medically based and practical treatment algorithm, as shown in Fig. 4 [114].

**Fig. 3:**
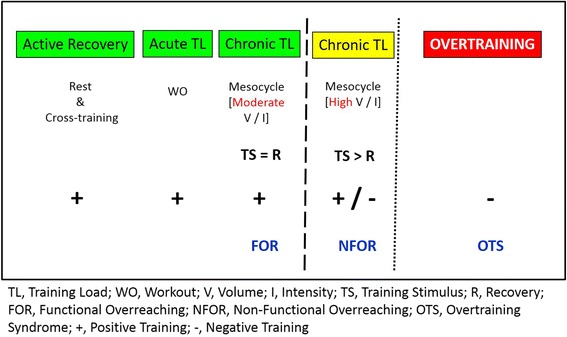
Overtraining continuum. Reproduced from Wilber et al. [114] with permission

**Fig. 4:**
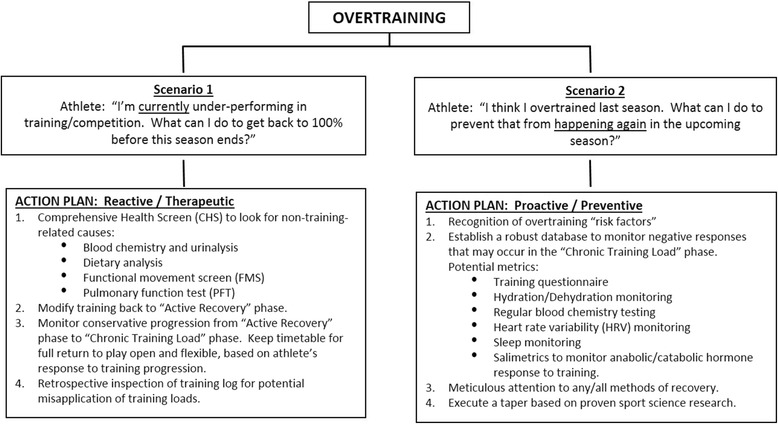
Overtraining treatment algorithm. Reproduced from Wilber et al. [114] with permission

### Child, Adolescent, and Masters Swimmers

The young and Masters athlete represent two special populations of swimmers that present unique conditions in their medical care. Swimming is an excellent activity for the young athlete that not only promotes physical activity but also helps build psychosocial and academic skills. For the Masters athlete, swimming improves physical performance without impact on the weight bearing joints that may contribute to degenerative joint disease. In addition, regular physical activity and training regimens often delay and blunt the aging-related declines in aerobic and anaerobic fitness. However, swimming can have a negative impact on the health and safety of both groups.

Early sports specialization, OT, and burnout are various conditions that commonly affect the young swimming athlete. Increasing desires by parents, guardians, coaches, and young athletes often drive more intensive and earlier training schedules. Athletes as young as 11 years are swimming >25 h per week in the pool. Training schedules often include swimming several hours in the morning, going to school, and then swimming several hours after school. Some young athletes are swimming 20–30 km per week, which has an aerobic equivalent to running 100 km. Many young athletes swim all year round, with only 1 day off of swimming per week. This intensive training schedule, combined with early sports specialization, can lead to a significant risk for overuse injuries as well as mental and physical burnout. Female and male athletes are at risk for the RED-S if they are not consuming enough calories from both daily and exercise activities. Young athletes that exhibit decreasing performance emotional or behavior changes, or have prolonged recovery from common overuse injuries may be experiencing OT or burnout. Sports medicine physicians are encouraged to counsel their young athletes to take at least 1–2 days off from sports per week, not to specialize in one sport until late adolescence, and to spend 2–3 calendar months participating in another sport [118–121]. Additionally, young athletes should not spend more hours training than their chronological age per week [121]. The IOC recently published recommendations on youth athlete development [122].

Participation in swimming by the Masters athlete has become an increasingly popular sport, with the United States Masters Swimming (USMS) as the governing body [123]. Swimming offers the Masters athlete the opportunity to improve their physical conditioning and delay the most common chronic diseases of the aging population which include arthritis, hypertension, and heart disease. The physiology of aging puts the Masters athlete at a unique disadvantage in comparison to the younger athlete [121, 124]. Most importantly are the changes in the cardiovascular (decreased maximal heart rate, decreased cardiac output), pulmonary (decreased total lung capacity, decreased maximal oxygen uptake), and musculoskeletal (decreased strength and bulk, decreased flexibility) system. Sex-related performance differences are less significant among female elite swimmers with advanced age in comparison to counterpart marathon runners [125]. Medications that interfere with blood pressure and heart rate may also influence performance and safety for the Masters athlete. Masters athletes are at risk for the same overuse injuries seen in the young athlete; however, the recovery and rehabilitation from these injuries may be prolonged. Thus, it is important to recognize these injuries early to prevent further progression. Rehabilitation from injuries is even more critical for the Masters athlete to prevent more rapid loss of flexibility, strength, and endurance than seen in younger athletes [121].

### Near-Drowning and Drowning

Drowning is the major cause of death worldwide (449,000 in 2000) and the second leading cause of unintentional death in children and adolescents in the USA [126, 127]. Children should be taught to swim. Children younger than 5 years of age should be supervised all the time, as instruction in younger children may lead to a false sense of security [127]. Additional counseling techniques include never swimming alone, swimming with adult supervision, the use of an approved personal floatation device, and the risks of the consumption of alcohol and other drugs [127]. A lifeguard or someone familiar with cardiopulmonary resuscitation (CPR) should be present during each practice and competition [127].

### Open-Water Swimming

The unique environmental conditions of open-water swimming inherently belie the potential injuries. In addition, marathon swimming, with some competitions greater than 25 km, adds interesting physiologic conditions that can affect competitors [11]. With training, most open-water swimmers do longer-distance workouts, also increasing potential for overuse injuries.

As with pool swimming, the most likely cause of injury is overuse of the shoulder in training. The highest in-competition injury risk of any aquatic discipline at the FINA World Championship was reported to be open-water swimming [23]. The risk of an in-competition injury was 57.7/1000 starts of female athletes with most of those being contact injuries with another athlete resulting in contusion.

Environmental conditions such as water/air temperature, water quality, and aquatic fauna and flora create the most unique aspect of open-water swimming [125]. Poor water quality can result in gastrointestinal illness. This can cause dehydration especially given the inability of athletes to frequently hydrate during races. Animal exposure (e.g., jellyfish, rays, fish, sharks, dolphin, seals, snakes) can all be problematic [128]. Because swimmers will be in the water for such long periods, prevention of chaffing and rubbing of the skin is important [128]. Exhaustion and fatigue can possibly exacerbate pre-existing medical conditions. This can be notable in both recreational as well as elite swimmers.

Lastly, the team and swimmer are reliant on training and race support. On most open-water courses, the swimmer is some distance from coaches and land-based medical personnel, relying on lifeguards and other, usually boat-based, observers. However, boats, including those of support crew and media covering events can unintentionally injure swimmers if in proximity. Communication between the athlete, coach, and support crew is usually minimal.

Keys to providing optimal medical care at open-water training and competitions include planning for the temperature, current, and other water conditions as well as endemic threats [128]. This can include providing hydration on course such as via boat supports. Preparing, briefing, and executing a medical response and evacuation plan are paramount.

## Conclusions

Swimming continues to draw athletes of all ages and competition levels. Traditionally, long hours and frequent practice sessions are common training programs among young and elite swimmers. This places swimmers at high risk for developing overuse injuries, nutrition deficits, and OT. Medical, coaching, and administrative staff, as well as athletes and parents, should be familiar with symptoms and signs of these conditions. More robust and methodologically sound studies are needed to help create comprehensive and evidence-based guidelines for taking care of swimmers.
